# Editorial

**Published:** 1874-08

**Authors:** 


### Editorial.

### *"One More Unfortunate."*

The notorious "Dr." Earll once again, for the twentieth time (more or less) has the opportunity to contemplate the outside world, from the inside (right side) of the jail-bars. A neighbor of his once earned the soubriquet of the "great unshot." Might not Earll, with still greater propriety, be designated as the great unhanged? As the case stands now, he is certainly the most persecuted individual that ever attempted to obliterate the evidences of human frailty with a bougie. Three times within less than six months, and how many times before that we have forgotten, have the minions of the law seized upon their victim, dragged him before coroners' juries, charged him with murder, double murder, for against such, single murders are not counted,—locked him behind prison bars, and what? — set him free, in order to play over again the same farce.

Years ago this man was charged by his own wife, under oath, with the crime of abortion, and with the added enormity of burning his own aborted child, piece-meal, in the stove. Here were two possible crimes suggested, of which one must necessarily have been committed, murder or perjury. If the woman told the truth, the man should have been hanged for murder; if she lied, she should have been sent to the penitentiary for perjury. Does the law encourage these crimes? it would seem so, since here is one or the other positively proven, and yet the allotted punishment is withheld.

And now, in the present case, a perfect net-work of crime is displayed: Adultery, perjury upon perjury, shamelessly admitted before the coroner's jury; murder, double murder, more perjury, flagrant violations of known laws, more perjury—and yet out of all this crime no specific act will be fixed upon any one, and no one will be punished. The actors will be turned loose to prey upon society, and juries will try to stifle their consciences with the hard-strained plea that they have "well and truly tried the case," "so help them God," which last prayer should be rather "God help them."

---

[NOTE.—We are indebted to the kindness of Prof. E. L. Holmes for the original of the following circular, which we translate entire for the benefit of our readers.]

***Periodical International Congress of the Medical Sciences.***
*Fourth Session. Brussels*, 1873.

STATUTES AND PROGRAMME.

In order to carry out the decision made by the Medical Congress of Vienna, on the 6th of September last, designating the city of Brussells as the locality of the next meeting of the Periodical

International Congress of the Medical Sciences, a Committee of Organization was appointed. This committee—composed of M. Vleminckx, President of the Royal Academy of Medicine of Belgium, President; M. Deroubaix, Vice-President (acting); MM. Bellefroid and Crocq, Ex-Vice-Presidents, members; and M. Warlomont, Secretary—has arranged the statutes and programme of the meeting as follows:

ART. 1. An International Medical Congress shall meet at Brussels, on the 19th of September, 1875, under the auspices of the Government.

ART. 2. The Congress, exclusively scientific, shall remain in session one week.

ART. 3. The Congress shall be composed of members of the medical profession, both national and foreign, who shall have forwarded their credentials of membership to the General Secretary. They shall not be subjected to any contribution, and shall alone be allowed to participate in the discussions.

ART. 4. The work of the Congress shall be divided into five sections, *i. e.:* 1st, Medicine, Surgery and Obstetrics; 2nd, Military Surgery (ambulance supply and service); 3rd, Hygiene; 4th, Ophthalmology; 5th, Pharmacology.

ART. 5. Upon the receipt of their certificates, members will be enrolled in the sections with which they may desire to unite. Any member may also be enrolled in several sections. Each of the sections shall choose its President, two Vice-Presidents, and a Secretary.

ART. 6. The Congress shall meet twice daily, from 10 A. M. to 1 P. M., for work in the sections; in the afternoon, from 1.30 to 5, for that of the general meeting.

ART. 7. Reporters, designated in advance by the committee, will state to the sections the subjects which shall have been assigned to them. This statement will terminate with provisional conclusions, which will have been published several months in advance of the meeting of the Congress, and which the sections shall examine in the order adopted by agreement. This work finished, they (the sections) will devote the remainder of the time to the reception of communications referring to the specialty of each, or outside of the programme. The definite conclusions voted by the sections will be next submitted by reporters, selected by them, to the sanction of the General Assembly.

Art. 8. The sessions of the General Assembly shall be devoted : 1st, To the communication of investigations directed to subjects outside of the programme ; 2nd, To the discussions of reports—in the order of their presentation—and, at the proper time, to voting, by the Congress, upon the conclusions proposed by the sections.

Art. 9. Members desiring to make communications upon subjects foreign to those upon the programme, should notify the General Secretary at least one month before the opening of the Congress. The Committee will designate the appropriate time for the presentation of such communications, and also the order in which they shall be presented. The time devoted to each speaker will be limited to a maximum of twenty minutes. This restriction not being applicable to reporters.

Art. 10. At the first session the Congress shall nominate its Administrative Staff, which shall consist of a President, two acting Vice-Presidents, an indefinite number of honorary Vice-Presidents, a General Secretary, and Recording Secretaries.

Art. 11. All papers read before the Congress shall be laid upon the table. The Committee of Organization, which will resume its functions after the session and proceed to publish the transactions of the Congress, will decide upon the total or partial, or non-insertion of each in the report.

Art. 12. Whilst the sessions will be conducted in French, members will be permitted to express themselves in other languages. In such cases, if it be so desired, an abstract of the sense of their remarks will be translated by one of the members present at the meeting.

Art. 13. The President will regulate the sessions and the debates in accordance with the forms generally adopted in deliberative assemblies. He will determine the order of the day, after consultation with the other officers.

Art. 14. Medical students may receive cards of admission, but will not be admitted to participation in the discussions.

The Committee is now occupied in the selection of subjects for the formation of the Programme. They will be glad to receive, from whatever direction they may come, communications which may be addressed to them on this subject, and will consider them in the constitution of its Programme, which will be published in the medical journals for the month of January next, with the provisional

CONCLUSIONS OF THE COMMITTEE. COPIES WILL BE ADDRESSED TO MEMBERS WHO MAY APPLY FOR THEM.

*The Committee requests editors of medical journals throughout the world to kindly give the greatest and speediest publicity possible to this communication.*

(Signed) VLEMINCKX, *President.*
WARLOMONT, *General Secretary.*

BRUSSELLS, April 15, 1874.

All communications must be addressed to M. le Dr. Warlomont, 152 Rue Royale a Bruxelles.

---

### *Married.*

At Indianapolis, July 2, by Rev. Robert Collyer, Miss MELLIE R. MANLOVE, daughter of the late Absalom Manlove, Esq., of that city, and Dr. R. L. REA, of Chicago.

